# Prevalence of Epiretinal Membrane among Subjects in a Health Examination Program in Japan

**DOI:** 10.3390/life11020093

**Published:** 2021-01-27

**Authors:** Hiroshi Shimizu, Ryo Asaoka, Takashi Omoto, Yuri Fujino, Shingo Mitaki, Keiichi Onoda, Atsushi Nagai, Shuhei Yamaguchi, Masaki Tanito

**Affiliations:** 1 Department of Ophthalmology, Shimane University Faculty of Medicine, Izumo 693-8501, Japan; hshimizu@med.shimane-u.ac.jp; 2 Department of Ophthalmology, Seirei Hamamatsu General Hospital, Hamamatsu 430-8558, Japan; ryoasa0120@googlemail.com (R.A.); fjnyr38@gmail.com (Y.F.); 3 Department of Ophthalmology, The University of Tokyo, Tokyo 113-0033, Japan; takashiomoto32@gmail.com; 4 Department of Neurology, Shimane University Faculty of Medicine, Izumo 693-8501, Japan; shingomi@med.shimane-u.ac.jp (S.M.); anagai@med.shimane-u.ac.jp (A.N.); yamagu3n@med.shimane-u.ac.jp (S.Y.); 5 Faculty of Psychology, Otemon Gakuin University, Ibaraki 567-8502, Japan; k-onoda@otemon.ac.jp

**Keywords:** epiretinal membrane, prevalence, aging, hypertension, hyperlipidemia

## Abstract

The prevalence of an epiretinal membrane (ERM) was elucidated using a dataset from a health examination program database in Japan. From the cohort database, 5042 eyes of 2552 subjects were included. The presence of an ERM, cellophane macular reflex (CMR), or preretinal macular fibrosis (PMF) was detected using color fundus photographs, and crude and age-standardized prevalence were obtained. To further assess the possible risk factors of ERM, background parameters were compared between ERM+ and − groups, and multiple logistic regression analysis was performed. ERM was detected in fundus photographs of 275 eyes (eye-based prevalence of 5.5%) from 217 subjects (subject-based prevalence of 8.5%). CMR was detected in 169 eyes (3.4%) of 138 subjects (5.4%), and PMF was detected in 106 eyes (2.1%) of 97 subjects (3.8%). By univariate analyses, compared with ERM− eyes or subjects, higher Scheie’s H grade (*p* < 0.0001), S grade (*p* < 0.0001), and glaucoma prevalence (*p* = 0.0440) were found in ERM+ eyes, and older age (*p* < 0.0001), more frequent histories of hypertension (*p* = 0.0033) and hyperlipidemia (*p* = 0.0441), and more frequent uses of medication for hypertension (*p* = 0.0034) and hyperlipidemia (*p* = 0.0074), shorter body height (*p* = 0.0122), and higher systolic blood pressure (*p* = 0.0078), and thicker intimal medial thickness (*p* = 0.0318) were found in ERM+ subjects. By multivariate analyses, older age (*p* < 0.0001, estimate = 0.05/year) was the only significant factor of ERM prevalence. Age-standardized prevalence of ERM was calculated to be 2.4%, 6.7%, and 13.3% for all ages, subjects older than 40 years, and subjects older than 65 years, respectively. We reported the prevalence of ERM and its subclasses in Japanese subjects. Since its prevalence is remarkably high in older subjects, an ERM can be seen as an important cause of visual impairment in Japan and in areas of the world where individuals live to an advanced age.

## 1. Introduction

An epiretinal membrane (ERM) is a sheet-like structure which develops on the inner surface of the neurosensory retina. ERM can be classified into idiopathic or secondary to other retinal pathologies including retinal breaks, retinal vein occlusion, diabetic retinopathy, uveitis, and other ocular inflammatory diseases [1]. ERM can be further classified into cellophane macular reflex (CMR), an early form of ERM, and preretinal macular fibrosis (PMF), a late phase of ERM.

The prevalence of ERM was investigated in several studies in the United States [2,3,4], Singapore [5,6,7], China [8,9,10,11], Korea [12,13], Australia [14,15,16,17], France [18], and Japan [19,20]. In these previous reports, prevalence varied between 2.2% in a Beijing study in rural China [10] and 28.9% in the United States [4]; thus, considerable variation in ERM prevalence across studies has been noted. In Japan, two major population-based studies have reported the prevalence of ERM; the prevalence was 4.0% in a Hisayama study [19] and 5.44% in a Funagata study [20].

To assess the reproducibility of ERM prevalence in the Japanese population, the current cross-sectional study evaluated the prevalence of ERM, CMR, and PMF among subjects involved in a health examination program in Japan. In addition, possible background factors associated with ERM prevalence were also assessed.

## 2. Subjects and Methods

### 2.1. Subjects

The Institutional Review Board of the Shimane University Faculty of Medicine approved this study (IRB No. 20190131-1), which was conducted according to the tenets of the Declaration of Helsinki. The IRB approval did not require each patient to provide written informed consent for publication; instead, the study protocol was posted at the study institutions to notify participants about the study. The cohort database included 6070 Japanese subjects who participated in a health examination system in the Shimane Institute of Health Science [21,22] from April 2005 to March 2019. When multiple visits were recorded in a subject, data of the oldest visit were collected. After the exclusion of 95 fundus photographs with poor image quality, we chose 5042 eyes of 2552 subjects from the database who had an interpretable color fundus photograph for at least one eye.

From the database, the following parameters were retrieved for the analyses; histories of systemic hypertension, hypertension medication, diabetes, diabetes medication, hyperlipidemia, hyperlipidemia medication, cardiovascular disease, stroke, smoking habit, age, sex, height, systolic blood pressure (sBP), diastolic blood pressure (dBP), body mass index (BMI), percent body fat, blood examination values, and mean intimal medial thickness (IMT) of both carotid arteries. The BMI was calculated as the body weight (kg) divided by the square of the body height (m). The blood examination included measurement of the total protein (TP), albumin, albumin/globulin ratio (A/G), bilirubin, aspartate aminotransferase, alanine aminotransferase (ALT), guanosine triphosphate, alkaline phosphatase, total cholesterol, triglyceride, high-density lipoprotein (HDL) cholesterol, low-density lipoprotein (LDL) cholesterol, hemoglobin A1c (HbA1c), white blood cell count, red blood cell count, hemoglobin, hematocrit, platelet count, fibrinogen, blood urea nitrogen (BUN), creatinine, sodium (Na), potassium (K), chlorine (Cl), calcium (Ca), uric acid, and amylase. The IMT was measured by ultrasonography (HI VISION Avius, Hitachi, Ltd., Tokyo, Japan).

### 2.2. Detection of ERM Using Color Fundus Photographs

Experienced laboratory technicians recorded color fundus photographs by using a non-mydriatic fundus camera with 45 view-angle (before December 2012 using CR6-45NM, Canon, Tokyo, Japan, and after January 2013 using CR-2, Canon). As an initial step, one (MT) author reviewed all fundus photographs and picked-up the photographs with a possible presence of ERM. Simultaneously, hypertensive (H0 to H4) and sclerotic (S0 to S4) changes to the retinal vessel were classified according to the Scheie’s grading system [23]; the presence of glaucoma also was labeled according to the recommendations of the Japan Glaucoma Society Guidelines for Glaucoma [24]. As a second step, one author (HS) reviewed the chosen photographs, and confirmed the presence of ERM. Simultaneously, ERM was classified into CMR or PMF according to the previously reported grading of ERM [20,25]. CMR was defined as the presence of increased light reflex from the retinal inner surface without retinal fold formation, whereas PMF was defined as the presence of an opaque greyish appearance and/or retinal folds due to the presence of a fibrous membrane on the inner retinal surface [20,25]. The presence of fundus pathologies that can be possible causes of ERM formation was also assigned. Using these methods of ERM detection, the false positive rate (i.e., overdiagnosis) was thought to be very low. To assess the possible underdiagnosis of ERM during the initial step, 200 fundus photographs (100 photographs each for 2 different fundus cameras) that had not been assigned as ERM by the initial step were randomly selected, and were re-evaluated by one author (HS). As a result, no ERM was found in these 200 photographs, indicating that the false negative rate was 0%.

### 2.3. Statistical Analysis

All data analyses were performed using JMP Pro statistical software, version 14.2 (SAS Institute Japan, Tokyo, Japan) on a Macintosh personal computer. Continuous variables were expressed as the mean ± standard deviation. The eye- and subject-based prevalence of ERM, CMR, and PMF were calculated in total subjects. Subject-based prevalence of ERM was also calculated in each 5-year step age group; the age-standardized prevalence of ERM in all ages, ages older than 40 years, and ages older than 65 years was calculated based on the World Health Organization (WHO) standard age distribution [26]. For comparisons of various background parameters between ERM+ and − groups, an unpaired t-test was used for continuous variables and Fisher’s exact probability test or a G test was used for categorical variables. To further assess the possible risk factors of ERM, multiple logistic regression analysis was performed in which the presence or absence of ERM served as a dependent variable and the background parameters served as independent variables; among the background parameters, Na, K, Cl, and Ca were excluded because the data were missing for these parameters for more than 10% of the subjects. For model construction, a stepwise forward selection method with a minimal Bayesian information criterion stopping rule was chosen.

## 3. Results

ERM was detected in fundus photographs of 275 eyes (eye-based prevalence of 5.5%) from 217 subjects (subject-based prevalence of 8.5%) (Table 1). The Scheie’s H grade (*p* < 0.0001), S grade (*p* < 0.0001), and glaucoma prevalence (*p* = 0.0440) were significantly higher in eyes with ERM than those without ERM. Possible causes of ERM were found in five eyes (three branch retinal vein occlusion, one retinal macroaneurysm, and one pan-retinal photocoagulation) in the ERM+ group. CMR was detected in 169 eyes (3.4%) of 138 subjects (5.4%), and PMF was detected in 106 eyes (2.1%) of 97 subjects (3.8%). In ERM+ subjects, ERM was present in both eyes in 58 subjects (26.7%) and in one eye in 159 subjects (73.3%). CMR and PMF was found in both eyes in 31 subjects (14.3%) and 9 subjects (4.1%), respectively, while CMR and PMF was found in only one eye in 18 subjects (8.3%). Regarding the availability of fundus photographs, the rate of study inclusion of both eyes/one eye from a subject was not different between the ERM+ and − subject groups (*p* = 0.8163).

Through the univariate comparison of demographics and background factors, compared with ERM− groups, older age (*p* < 0.0001), more frequent histories of hypertension (*p* = 0.0033) and hyperlipidemia (*p* = 0.0441), and more frequent use of medication for hypertension (*p* = 0.0034) and hyperlipidemia (*p* = 0.0074), shorter body height (*p* = 0.0122), and higher sBP (*p* = 0.0078), and thicker IMT (*p* = 0.0318) were found in the ERM+ group, while sex, histories of diabetes, cardiovascular disease, and stroke, use of diabetes medication, smoking habit, body weight, BMI, and dBP were not different between ERM+ and − groups (Table 2).

**Figure 1:**
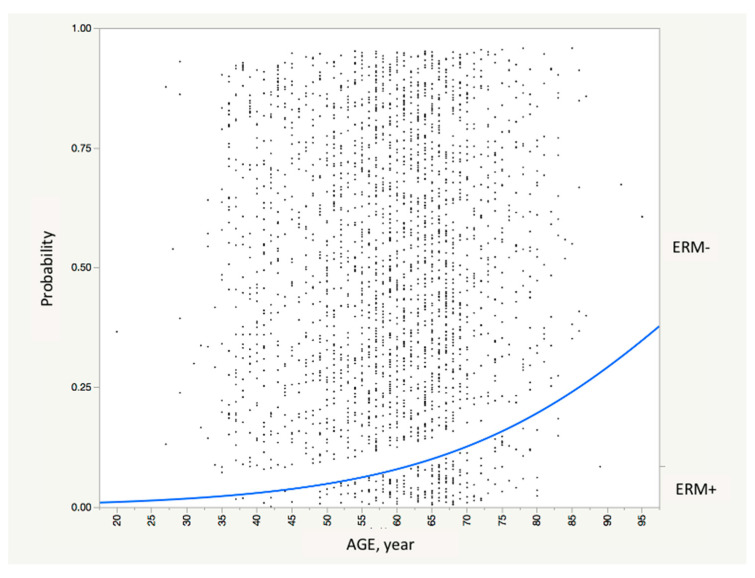
Logistic fit of presence and absence of ERM (subject-based) by subject age. The curve indicates the predicted probability of the presence of ERM (left *y*-axis) as a function of age (*x*-axis) (*p* < 0.0001). In this model, the parameter estimate is calculated to be 0.05/year (95%CI range, 0.04–0.07/year).

None of the 27 laboratory examination data compared were different between the ERM+ and – groups (Table 3). As shown the multivariate analysis, among the factors included in the model (all the factors listed in Table 2 and Table 3 were included into the model), older age (*p* < 0.0001, estimate = 0.05/year) was the only significant factor that was associated with ERM prevalence (Figure 1).

Finally, since age was revealed to be the critical factor for the presence of ERM, the WHO Standard age distribution was used to estimate the ERM prevalence. As a result, age standardized prevalence of ERM in our study was calculated to be 2.4% for all age groups, 6.7% for subjects older than 40 years, and 13.3% for subjects older than 65 years (Table 4 and Table 5).

## 4. Discussion

In this cross-sectional study, an ERM was found in 5.5% of eyes and in 8.5% of subjects among the subjects who received a health examination program in Japan. Previously, two major population-based studies reported ERM prevalence in Japan; the prevalence was reported to be 4.0% of subjects in a Hisayama study [19] and 5.44% in the right eye in a Funagata study [20]. Increasing age was consistently identified as a risk factor for ERM in this and most previous studies [1]. After age adjustment by WHO standard age distribution, the prevalence of 2.4% in all age groups was close to the age-adjusted prevalence of 2.8% in the Hiasayama study and slightly lower than the prevalence of 3.7% in the Funagata study [25]. In the reports from other countries, age-adjusted ERM prevalence was calculated to be 6.4% in the Beaver Dam Eye study (USA), 5.5% in the Blue Mountain Eye study (Australia), 3.5% in the Handan Eye Study (China), 7.6% in the Jiangning Eye study (China), 19.0% in the Los Angeles Latino Eye study (USA), 24.5% in the Multi-Ethnic Study of Atherosclerosis (USA), 9.3% in the Singapore Malay Eye study (Singapore), 13.0% in the Singapore Chinese Eye study, 8.8% in the Singapore Indian Eye study (Singapore), and 4.9% in the Visual Impairment Project (Australia) [25]; all of these studies used fundus photographs for the assessment of ERM as current reports. Collectively with the Funagata and Hisayama studies, ERM prevalence in Japan seems lower than Caucasians in western countries and Asians in Singapore.

Other than ethnicity and race, various factors such as gender, refractive error, and systemic conditions had been speculated to be associated with ERM, but have not been confirmed [1]. By univariate analysis, the presence of hyperlipidemia and use of anti-hyperlipidemia medication was associated with ERM. This is in line with the previous studies [4,14] including the Hisayama study [19]. The confounding effect of hyperlipidemia might explain the higher Scheie’s S grade in ERM eyes and thicker IMT in ERM subjects in this study. Findings regarding a possible association between hypertension or use of anti-hypertension medication and ERM is unique in the literature. Previously, narrowing of retinal vasculature was reported in eyes with ERM [6,7]; this coincided well with our observation of higher Scheie’s H grade in ERM eyes. Although retinal vasculature changes can be explained by traction of retinal vasculatures by ERM, our results suggest the possible link between hypertension, hypertensive changes in retina vessels, and ERM formation, but this remains to be elucidated. We found a higher prevalence of glaucoma in ERM eyes. By using optical coherence tomography (OCT) among subjects older than 75 years old, the prevalence of ERM was found to be higher in eyes with glaucoma than without glaucoma [27]. Prevalence of a retinal nerve fiber layer defect (RNFLD) was higher in eyes with ERM than without ERM, while optic disc cupping was equivalent between with and without ERM. Although we carefully excluded the non-glaucomatous, ERM-associated RNFLD [28], the association between glaucoma and ERM was still inconclusive in this study. The association between ERM and shorter body height also needs to be confirmed in future study. In the multivariate analysis, older age was the only significant factor that associated with ERM prevalence, thus we cannot exclude the possibility that the confounding effect of age might explain all the associations between ERM and the risk factors detected by the univariate analyses.

Myopia [29] and cataract surgery [2,3,16] were reported to be risk factors of ERM. Accordingly, a lack of data regarding refraction and ocular surgical history is a limitation of our study. We found possible causes of secondary ERM in only five ERM eyes, thus we believe that most of the ERMs in this study are idiopathic. However, more secondary ERM-related pathologies can be found if we examined the peripheral fundus or used OCT [18]. Participants who received a health examination might be different to the participants of a community-based study with respect to health consciousness, education, and income, thus different backgrounds might limit the direct comparison between our data and previously reported ERM prevalence derived from population-based studies. In our study, most of the participants recorded the fundus photographs, and <2% (95/5132 eyes) of the fundus photographs were excluded from the analyses, and thus the high response rate is a strength of our study.

## 5. Conclusions

In summary, we reported the prevalence of ERM and its subclasses in Japanese subjects. Based on the vital statistics of the Japanese government, the aging rate of the population aged 65 years old or older in Japan, i.e., 27.3% in 2016, is the highest in the world. Although the prevalence of ERM in Japan seems lower in general age groups than other countries/races, ERM can be an important cause of visual impairment in Japan, since its prevalence becomes remarkably high in older subjects. In our results, CMR was present in 61.3% (169/275 eyes) of ERM eyes, thus the requirement of surgical treatment is also expected to be increased. In the same context, we expect that the importance of ERM is going to increase in the future in areas of the world where individuals will live to an advanced age.

## Figures and Tables

**Table 1:** Summary of fundus photograph classification (5043 eyes of 2552 subject).

Parameters	ERM+	ERM−	*p*-Value
**Eye-based**			
ERM, n (%)	275 (5.5)	4767 (94.5)	
Scheie’s H grade			
mean ± SD	0.67 ± 0.60	0.46 ± 0.57	<0.0001 ^†^
95%CI	0.60–0.74	0.44–0.47	
Scheie’s S grade			
Mean ± SD	1.00 ± 0.55	0.64 ± 0.54	<0.0001 ^†^
95%CI	0.62–0.65	0.01–0.62	
Glaucoma			
yes, n (%)	26 (9.5)	301 (6.3)	0.0440 ^‡^
no, n (%)	249 (90.5)	4466 (93.7)	
Possible cause of ERM	3 BRVO		
	1 MA		
	1 PRP		
CMR, n (%)	169 (3.4)	4873 (96.6)	
PMF, n (%)	106 (2.1)	4936 (97.9)	
**Subject-based**			
Available photographs			
Both eyes, n (%)	213 (98.2)	2277 (97.5)	0.8163 ^‡^
One eye, n (%)	4 (1.8)	58 (2.5)	
ERM, n	217 (8.5)	2335 (91.5)	
Both eyes, n (%)	58 (26.7)		
One eye, n (%)	159 (73.3)		
CMR, n	138 (5.4)		
both eyes, n (%)	31 (22.5)		
one eye, n (%)	107 (77.5)		
PMF, n	97 (3.8)		
Both eyes, n (%)	9 (9.3)		
One eye, n (%)	88 (90.7)		
ERM Subject breakdown, n	217		
CMR in both eyes, n (%)	31 (14.3)		
CMR in one eye, n (%)	89 (41.0)		
CMR and PMF in one eye each, n (%)	18 (8.3)		
PMF in both eyes, n (%)	9 (4.1)		
PMF in one eye, n (%)	70 (32.3)		

*p*-values are calculated by un-paired t-test (^†^) and Fisher’s exact probability test (^‡^). ERM, epiretinal membrane; SD, standard deviation; CI, confidence interval; CMR, cellophane macular reflex; PMF, preretinal macular fibrosis; BRVO, branch retinal vein occlusion; MA, retinal macroaneurysm; PRP, panretinal photocoagulation.

**Table 2:** Comparison of demographic and systemic data between ERM+ and − subjects (subject-based analyses).

Parameters	ERM+	ERM−	*p*-Value
Age, years			
n	217	2335	
mean ± SD	64.3 ± 8.3	58.7 ± 11.0	<0.0001 ^†^
95%CI	63.2–65.5	58.2–59.1	
Sex			
male, n (%)	112 (51.6)	1293 (55.4)	0.3179 ^‡^
female, n (%)	105 (48.4)	1042 (44.6)	
Hypertension			
yes, n (%)	121 (56.3)	1025 (45.6)	0.0033 ^‡^
no, n (%)	94 (43.7)	1225 (54.4)	
Hypertension medication			
yes, n (%)	70 (33.3)	527 (24.0)	0.0034 ^‡^
no, n (%)	140 (66.7)	1670 (76.0)	
Diabetes			
yes, n (%)	19 (8.9)	216 (9.7)	0.8082 ^‡^
no, n (%)	194 (91.1)	2016 (90.3)	
Diabetes medication			
yes, n (%)	3 (1.5)	61 (2.9)	0.2527 ^‡^
no, n (%)	197 (98.5)	2015 (97.1)	
Hyperlipidemia			
yes, n (%)	111 (51.9)	997 (44.6)	0.0441 ^‡^
no, n (%)	103 (48.1)	1240 (55.4)	
Hyperlipidemia medication			
yes, n (%)	51 (23.9)	361 (16.4)	0.0074 ^‡^
no, n (%)	162 (76.1)	1840 (83.6)	
Cardiovascular disease			
yes, n (%)	48 (22.5)	477 (21.4)	0.7269 ^‡^
no, n (%)	165 (77.5)	1752 (78.6)	
Stroke			
yes, n (%)	8 (3.8)	41 (1.8)	0.0691 ^‡^
no, n (%)	205 (96.2)	2189 (98.2)	
Smoking habit			
yes, n (%)	23 (10.8)	336 (15.0)	0.1611 ^‡^
no, n (%)	131 (61.2)	1362 (60.9)	
past smoking, n (%)	80 (28.0)	540 (24.1)	
Height (cm)			
n	217	2332	
mean ± SD	159.7 ± 8.7	161.3 ± 9.1	0.0122 ^†^
95%CI	158.5–160.8	160.9–161.7	
Weight (kg)			
n	217	2331	
mean ± SD	59.3 ± 11.7	60.5 ± 11.4	0.1270 ^†^
95%CI	57.8–60.7	60.0–61.0	
BMI			
n	217	2331	
mean ± SD	23.1 ± 3.0	23.1 ± 3.2	0.9891 ^†^
95%CI	22.7–23.6	23.0–23.3	
Systolic BP (mmHg)			
n	216	2331	
mean ± SD	130.6 ± 17.1	127.3 ± 17.4	0.0078 ^†^
95%CI	128.3–132.9	126.6–128.0	
Diastolic BP (mmHg)			
n	216	2331	
mean ± SD	75.1 ± 11.7	73.8 ± 11.5	0.1049 ^†^
95%CI	73.5–76.7	73.3–74.2	
Mean IMT (mm)			
n	211	2201	
mean ± SD	0.92 ± 0.41	0.86 ± 0.36	0.0318 ^†^
95%CI	0.86–0.97	0.85–0.88	

*p*-values are calculated by unpaired t-test (^†^) and Fisher’s exact probability test or G test (^‡^). ERM, epiretinal membrane; BMI, body mass index; BP, blood pressure; IMT, intimal-medial thickness.

**Table 3:** Comparison of laboratory examination data between ERM+ and − subjects (subject-based analyses).

Parameters	ERM+	ERM−	*p*-Value
TP (g/dL)			
n	217	2329	
Mean ± SD	7.4 ± 0.4	7.4 ± 0.4	0.2520 ^†^
95%CI	7.4–7.5	7.4–7.4	
Albumin (g/dL)			
n	217	2334	
Mean ± SD	4.4 ± 0.2	4.4 ± 0.2	0.7648 ^†^
95%CI	4.4–4.5	4.4–4.4	
A/G			
n	217	2334	
mean ± SD	1.5 ± 0.2	1.5 ± 0.2	0.1146 ^†^
95%CI	1.5–1.5	1.5–1.5	
Total bilirubin (mg/dL)			
n	217	2334	
mean ± SD	0.8 ± 0.3	0.8 ± 0.3	0.5122 ^†^
95%CI	0.7–0.8	0.8–0.9	
AST (IU/L)			
n	217	2334	
mean ± SD	24.3 ± 9.4	24.7 ± 11.7	0.6108 ^†^
95%CI	23.0–25.6	24.2–25.2	
ALT (IU/L)			
n	217	2334	
mean ± SD	22.4 ± 12.8	24.0 ± 15.9	0.1277 ^†^
95%CI	20.6–24.1	23.4–24.7	
γGTP (IU/L)			
n	217	2334	
Mean ± SD	41.5 ± 65.3	44.0 ± 65.5	0.5982 ^†^
95%CI	32.8–50.3	41.3–46.6	
ALP (IU/L)			
n	217	2334	
mean ± SD	219.4 ± 63.3	219.9 ± 66.5	0.9176 ^†^
95%CI	211.0–227.9	217.2–222.6	
Total cholesterol (mg/dL)			
n	217	2332	
mean ± SD	213.5 ± 29.2	209.7 ± 33.3	0.0979 ^†^
95%CI	209.6-217.4	208.3-211.0	
Triglycerides (mg/dL)			
n	217	2334	
mean ± SD	125.5 ± 78.9	115.8 ± 76.9	0.0750 ^†^
95%CI	114.9–136.1	112.6–118.9	
HDL-C (mg/dL)			
n	217	2334	
mean ± SD	63.0 ± 16.2	63.3 ± 16.5	0.7547 ^†^
95%CI	60.8–65.1	62.7–64.0	
LDL-C (mg/dL)			
n	217	2329	
mean ± SD	122.6 ± 27.8	121.1 ± 30.8	0.4750 ^†^
95%CI	118.9–126.3	119.8–122.3	
HbA1c (%)			
n	217	2329	
mean ± SD	5.5 ± 0.5	5.5 ± 0.7	0.4757 ^†^
95%CI	5.4–5.6	5.4–5.5	
WBC (×10^2^/mL)			
n	217	2334	
mean ± SD	57.6 ± 15.5	57.0 ± 15.3	0.5927 ^†^
95%CI	55.6–60.0	56.4–57.7	
RBC (×10^4^/mL)			
n	217	2333	
mean ± SD	461.8 ± 42.5	464.8 ± 42.0	0.3188 ^†^
95%CI	456.1–467.5	463.1–466.5	
Hemoglobin (g/dL)			
n	217	2334	
mean ± SD	14.3 ± 1.4	14.4 ± 1.4	0.2525 ^†^
95%CI	14.1–14.5	14.3–14.5	
Hematocrit (%)			
n	217	2334	
mean ± SD	43.0 ± 3.5	42.9 ± 3.7	0.6541 ^†^
95%CI	42.5–43.5	42.7–43.0	
Platelet (×10^4^/mL)			
n	217	2334	
mean ± SD	22.3 ± 5.4	22.9 ± 6.1	0.2134 ^†^
95%CI	21.6–23.1	22.6–23.1	
Fibrinogen (mg/dL)			
n	210	2285	
mean ± SD	288.7 ± 60.3	287.2 ± 60.0	0.7316 ^†^
95%CI	280.5–296.9	284.7–289.6	
BUN (mg/dL)			
n	217	2333	
mean ± SD	15.0 ± 3.8	14.5 ± 3.8	0.1107 ^†^
95%CI	14.5–15.5	14.4–14.7	
Creatinine (mg/dL)			
n	217	2334	
mean ± SD	0.73 ± 0.17	0.74 ± 0.18	0.2776 ^†^
95%CI	0.71–0.75	0.74–0.75	
Na (mEq/L)			
n	200	1849	
mean ± SD	142.0 ± 1.9	141.9 ± 1.9	0.8357 ^†^
95%CI	141.7–142.2	141.9–142.0	
K (mEq/L)			
n	200	1849	
mean ± SD	4.1 ± 0.3	4.1 ± 0.3	0.2572 ^†^
95%CI	4.1–4.2	4.1–4.2	
Cl (mEq/L)			
n	200	1849	
mean ± SD	103.2 ± 2.1	1033 ± 2.4	0.5761 ^†^
95%CI	102.9–103.5	103.2–103.4	
Ca (mg/dL)			
n	199	1847	
mean ± SD	9.3 ± 0.3	9.3 ± 0.3	0.7528 ^†^
95%CI	9.3–9.4	9.3–9.4	
Uric acid (mg/dL)			
n	216	2334	
mean ± SD	5.2 ± 1.2	5.3 ± 1.3	0.1713 ^†^
95%CI	5.0–5.3	5.2–5.4	
Amylase (IU/L)			
n	216	2333	
mean ± SD	84.6 ± 26.8	80.9 ± 27.5	0.0566 ^†^
95%CI	81.0–88.2	79.7–82.0	

*p*-values are calculated by unpaired t-test (^†^). ERM, epiretinal membrane; TP, total protein; A/G, albumin/globulin; AST, aspartate aminotransferase; ALT, alanine aminotransferase; γGTP, guanosine triphosphate; ALP, alkaline phosphatase; HDL-C, high-density lipoprotein cholesterol; LDL-C, low-density lipoprotein cholesterol; HbA1c, glycosylated hemoglobin A1c; WBC, white blood cell; RBC, red blood cell; BUN, blood urea nitrogen; Na, sodium; K, potassium; Cl, chlorine; Ca, calcium.

**Table 4:** Eye-based prevalence of ERM stratified by age (5-year step).

Age, Years	ERM+, n	ERM-, n	Prevalence, %
0–4	0	0	0
5–9	0	0	0
10–14	0	0	0
15–20	0	0	0
20–24	0	2	0
25–29	0	14	0
30–34	0	22	0
35–39	2	254	0.8
40–44	4	316	1.3
45–49	4	327	1.2
50–54	14	536	2.5
55–59	41	854	4.6
60–64	54	939	5.4
65–69	91	872	9.4
70–74	32	329	8.9
75–79	24	196	10.9
80–84	8	80	9.1
85–89	1	23	4.2
90–94	0	2	0
95–100	0	1	0
100–	0	0	0
Total	275	4767	5.5

**Table 5:** Age-standardized subject-based prevalence of ERM.

Age, Years	ERM+, n	ERM−, n	Prevalence, %	WHO Standard Age Distribution, % (/Total) *	Age-Standardized Prevalence, % (/Total)	Age-Standardized Prevalence (>40 Years), % (/Total)	Age-Standardized Prevalence (>65 Years), % (/Total)
0–4	0	0	0	8.86	0.00		
5–9	0	0	0	8.69	0.00		
10–14	0	0	0	8.60	0.00		
15–20	0	0	0	8.47	0.00		
20–24	0	1	0	8.22	0.00		
25–29	0	7	0	7.93	0.00		
30–34	0	11	0	7.61	0.00		
35–39	2	126	1.6	7.15	0.11		
40–44	4	157	2.5	6.59	0.16	0.47	
45–49	4	162	2.4	6.04	0.15	0.42	
50–54	13	263	4.7	5.37	0.25	0.73	
55–59	32	419	7.1	4.55	0.32	0.93	
60–64	42	459	8.4	3.72	0.31	0.90	
65–69	72	418	14.7	2.96	0.43	1.26	5.25
70–74	24	161	13.0	2.21	0.29	0.83	3.46
75–79	17	95	15.2	1.52	0.23	0.67	2.79
80–84	6	43	12.2	0.91	0.11	0.32	1.35
85–89	1	11	8.3	0.44	0.04	0.11	0.44
90–94	0	1	0	0.15	0.00	0.00	0.00
95–100	0	1	0	0.04	0.00	0.00	0.00
100–	0	0	0	0.05	0.00	0.00	0.00
Total	217	2335	8.5	100	2.4	6.7	13.3

* Data are adopted from Ahmad OB et al., Age Standardization of Rates: A New WHO Standard, GPE Discussion Paper Series No 31., EIP/GPE/EBD World Health Organization 2001. ERM, epiretinal membrane.

## Data Availability

Data underlining this study is available upon reasonable request to corresponding author.
